# A merged molecular representation learning for molecular properties prediction with a web-based service

**DOI:** 10.1038/s41598-021-90259-7

**Published:** 2021-05-26

**Authors:** Hyunseob Kim, Jeongcheol Lee, Sunil Ahn, Jongsuk Ruth Lee

**Affiliations:** Center for Computational Science Platform, Korea Institute of Science and Technology Information, Daejeon, 34141 Republic of Korea

**Keywords:** Cheminformatics, Computer science

## Abstract

Deep learning has brought a dramatic development in molecular property prediction that is crucial in the field of drug discovery using various representations such as fingerprints, SMILES, and graphs. In particular, SMILES is used in various deep learning models via character-based approaches. However, SMILES has a limitation in that it is hard to reflect chemical properties. In this paper, we propose a new self-supervised method to learn SMILES and chemical contexts of molecules simultaneously in pre-training the Transformer. The key of our model is learning structures with adjacency matrix embedding and learning logics that can infer descriptors via Quantitative Estimation of Drug-likeness prediction in pre-training. As a result, our method improves the generalization of the data and achieves the best average performance by benchmarking downstream tasks. Moreover, we develop a web-based fine-tuning service to utilize our model on various tasks.

## Introduction

Drug discovery has traditionally been a very expensive research field. There are lots of candidates to validate and the process of validation is significantly complex and long. Recently, deep learning^1–3^ is utilized to increase efficiency and accelerate the overall process in drug discovery such as molecular property prediction, virtual screening, etc. However, there are still some obstacles to overcome to apply it to the actual industry. Despite the presence of a tremendous number of molecules, labeled datasets are scarce. The characteristics of the dataset cause limitations such as overfitting and unstable model performance in evaluating models.

Molecules can be converted to various kinds of data representations. Traditionally, fingerprint^4,5^ and descriptors are used as input features in constructing models. The molecular fingerprint is a popular method representing substructures. It encodes the structure of a molecule by using a hash function. Machine learning models use them as features with additional descriptors such as Partition coefficient (logP), Hydrogen Bond Acceptors (HBA), Hydrogen Bond Donors (HBD), Polar Surface Area (PSA), etc. However, fingerprints reveal limitations caused by the hash function in predicting properties. For solving those limitations, a neural networks-based fingerprint appears. Seq2seq fingerprint^6^ learns molecular embedding with recurrent neural networks (RNN).

Simplified Molecular-Input Line-Entry System (SMILES) is a text representation of molecules in a single line. As it is sequential and composed of text, methods inspired by NLP such as word embedding^7^ and RNN^8,9^ have been proposed. Mol2Vec^10^ is a molecular representation inspired by word2vec. It overcomes the drawbacks of fingerprint such as bit collisions. Smiles2Vec^9^ learns a representation of a molecule using RNN on SMILES tokens. It uses linear embedding on SMILES characters and predicts molecular properties using outputs of RNN. Convolutional Neural Networks (CNN)^11,12^ also work effectively on molecular properties prediction. However, there is a limitation that SMILES string cannot perfectly cover the chemical structures and coordinates of molecules. It causes performance degradation in training the model when a specific structure is not identified.

Self-supervised learning^13–15^ which does not need labeled data for training shows performance improvements in most tasks of natural language processing (NLP) by pre-training the model such as Transformer^16^. Those methods build a huge language model that can be utilized for various tasks using the pre-training. It demonstrates a generalized model can be constructed with large-scale data and fine-tuning works on downstream tasks. Pre-training approaches also appear to solve molecular properties predictions in drug discovery. SMILES-Transformer^17^ extracts latent vectors from the output of the encoder and uses it as a fingerprint of a molecule. It is superior to others in small-data settings. SMILES-BERT^18^ applies a pre-training task of BERT^14^ on SMILES and shows improved performance in several prediction tasks. Molecule Attention Transformer^19^ (MAT) unifies atomic features with structural information and augments attention weights with graph structure and distance. Those works demonstrate pre-training on the Transformer is valid on molecular data. Pre-training strategies on Graph Neural Networks^20^ (GNN) are also emerging. Context prediction and supervised attribute prediction are designed to train the model in both node-level and graph-level. They help to predict substructures and neighborhoods of nodes on molecule data. Overall researches show that well-designed pre-training strategies can reflect general characteristics of molecule data with improving overall performance on downstream tasks.

In this paper, we propose a new self-supervised method for pre-training named CHEM-BERT. We design the pre-training method that learns to contain chemical contexts in SMILES to overcome the limitations of SMILES. Our method is based on masking and recovery pre-training task that predicts masked tokens of SMILES using a Transformer. Additionally, we design the matrix embedding layer which can learn structures of molecules and add a Quantitative Estimation of Drug-likeness (QED) value prediction task in the pre-training step. The matrix embedding layer complements the connectivity of molecules which can be lost in SMILES. The QED prediction task helps to infer descriptors that are composing QED and solving downstream tasks. As a result, our model learns a merged SMILES-based representation containing chemical contexts of molecules in the pre-training step.

We perform benchmarking on 10 molecule datasets from MoleculeNet^21^. Each downstream task is performed by fine-tuning the pre-trained model. Our pre-training method improves the generalization of molecules compared with various representations. We demonstrate our model achieves the best average performance on both classification and regression tasks. Finally, we develop a fine-tuning service on the web-based server. It can learn various molecular properties prediction tasks using a pre-trained model. Users can easily get stable and high-performance results without constructing task-specific architectures.

## Methods

### CHEM-BERT: a merged molecular representation learning

**Figure 1:**
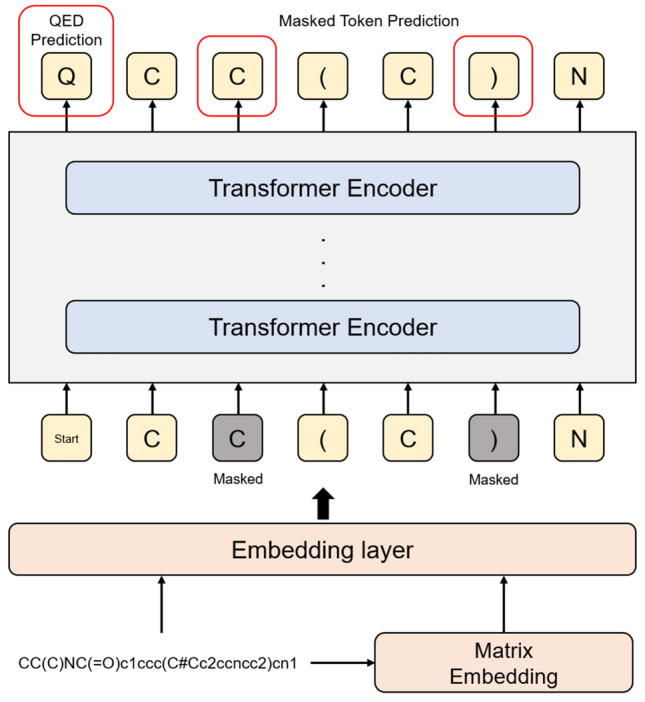
The overview of our pre-training methods and model. The input SMILES is converted to input vectors via embedding layer. The red boxes are targets of pre-training among the outputs of Transformer. The fine-tuning uses the same architectures except output layers.

Our self-supervised method for learning merged molecular representation is based on pre-training tasks and the architecture of BERT. For enhancing SMILES-based representations, we propose a matrix embedding layer and QED prediction task. The overall architecture is shown in Fig. 1. The adjacency matrix of input SMILES goes to the matrix embedding layer to produce a matrix embedding vector. After that, input SMILES is split into tokens and converted to a token embedding vector in the embedding layer. Input tokens are fed to the Transformer including masked tokens. The outputs of the Transformer consist of a QED value and recovered input tokens. Finally, the model performs predictions on masked tokens and QED simultaneously.

The Transformer is an encoder–decoder structure which can process the sequential input data. We use only Transformer-encoder in constructing the model. The encoder mainly consists of self-attention blocks and feed-forward networks. The self-attention means a mechanism relating different positions of a single sequence to compute a representation of the same sequence. The self-attention block has a Multi-Head Attention layer and computes attention weights as followed:

1 $$\begin{aligned}&A(Q,K,V)=softmax\left( \frac{QK^{T}}{\sqrt{d_{k}}}\right) V\end{aligned}$$

2 $$\begin{aligned}&Q=HW^{Q}, K=HW^{K}, V=HW^{V}, \end{aligned}$$

where $Q$ is the query matrix, $K$ is the key matrix, $V$ is the value matrix and $d_{k}$ is the dimension of queries and keys. $Q$, $K$, and $V$ are the same sequential input for computing the self-attention. $Q,K,V$ are a multiplication of input hidden state $H$ and the weight matrix $W^{Q},W^{K},W^{V}$. The softmax function is applied to obtain the attention weights. It is called Scaled Dot-Product Attention. Multi-Head Attention computes the value of several Scaled Dot-Product Attention layers in parallel. A fully connected feed-forward network is connected with the output of the self-attention block. This process is repeated as the number of layers.

The masked language model which is used in BERT predicts masked words for the given sentence. It is a self-supervised method in NLP using unlabeled data. We apply it to predict masked tokens of given SMILES from unlabeled molecular data. For masking, we tokenize the SMILES string and randomly choose 15% on all tokens of input data. We replace a chosen token to <MASK> with 80%. For the rest, it is replaced with another token in the dictionary with 10% or unchanged with 10%. This process is designed to prevent overfitting in pre-training.

**Figure 2:**
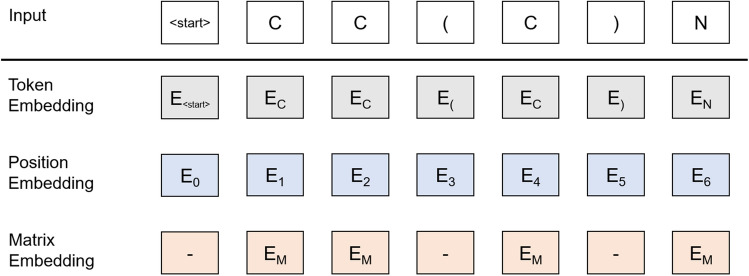
The input representation of SMILES. The input is the sum of the token embeddings, the position embeddings, and the matrix embeddings. The matrix embedding is only applied to atomic tokens.

However, SMILES is insufficient to represent the connectivity and substructures of molecules. Therefore, we design the matrix embedding layer to complement structural information as followed:

3 $$\begin{aligned} E(A)=W_{e}AW_{a}+b, \end{aligned}$$

where $W_{a}$ is the weight matrix for an adjacency matrix $A$, $W_{e}$ is the weight vector for generating an embedding token and $b$ is the bias vector. The matrix embedding layer learns the adjacency matrix of molecules and produces a vector for embedding. The adjacency matrix is useful information in prediction, but adding a matrix embedding vector on unrelated tokens disturbs training. Therefore, the matrix embedding vector produced from this layer is only applied to atomic tokens which are related to an adjacency matrix. As a result, the input SMILES is converted to the sum of token embedding, position embedding, and matrix embedding shown as Fig. 2. Position embedding notifies the sequence of input data. The start and end token are included in the input data to distinguish the length of SMILES more clearly.

BERT uses two sentences as input data and performs the next sentence prediction task. It helps BERT to understand the context of documents. Similarly, our model performs QED^22^ prediction as a second pre-training task to learn the properties of molecules. QED indicates the drug-likeness of molecules and reflects molecular properties such as logP, molecular weight, the number of aromatic rings, topological polar surface area, rotatable bonds, etc. It also reflects the underlying distribution of molecular properties and is calculated as a value between 0 to 1 considering the properties of the molecule as followed:

4 $$\begin{aligned} QED=\exp \left( \frac{1}{n}\sum _{i=1}^{n}\ln {d_{i}}\right) , \end{aligned}$$

where $d_{i}$ is a desirability function of each property. We expect the model constructs a logic that can infer properties and descriptors of the molecule during pre-training. By predicting the QED value, the model can predict descriptors and merge those properties in the output representation. We apply a linear classifier on the first output of the Transformer for prediction. In fine-tuning, this mechanism helps to predict descriptors and solving downstream tasks.

Additionally, we develop the service to utilize our pre-trained model on new datasets. It can accelerate the model development process with a user-friendly web-based interface. The service is operated with the following components.

- Data submission: Users can submit a prepared dataset and select the task type.
- Fine-tuning: Begin the fine-tuning with the pre-trained model. The result of training will be delivered to the viewer.
- Result viewer: The viewer shows the result of training including performances, graphs and details of the model.

We will address contributions of the service in Results and Discussion.

### Experimental settings

In pre-training, we use 9 million unlabeled molecules selected from ZINC^23^ database. For QED prediction, we compute the QED value on molecules to use it as a label. The range of QED value is 0.009–0.948. We test the effect of pre-training data size changing from 2 to 9 million while using validation set size as 10,000. We see that the validation performance gets higher as the training data size increases. The model which used 9 million data records 94.4% accuracy on masked tokens prediction and 0.02 Mean Absolute Error (MAE) on QED prediction. Therefore, we decide to use 9 million data for pre-training. We use Transformer Encoder with 8 layers and 16 attention heads. The activation function used in the Transformer is Gaussian Error Linear Units (GELU)^24^. The maximum input sequence length is 256. The input embedding vector and hidden units of the Transformer are 1024 dimensions. We do not apply dropout on the entire model which causes underfitting in this work. We use Adam^25^ optimizer with the scheduled learning rate as followed:

5 $$\begin{aligned} learning\_rate=0.1 \cdot d^{-0.5}_{model} \cdot min(step\_num^{-0.5}, step\_num \cdot warmup\_steps^{-1.5}). \end{aligned}$$

We use 10,000 as warm-up steps. The loss function for pre-training is the sum of Mean Squared Error and Cross-entropy. Mean Squared Error is used to train the QED value prediction and Cross-entropy is used to train the masked token prediction.

**Table 1:** Information of dataset from MoleculeNet.

Dataset	Tasks	# Molecules	Metric
BBBP	1	2039	ROC–AUC
Tox21	12	7831	ROC–AUC
ToxCast	617	8575	ROC–AUC
SIDER	27	1427	ROC–AUC
ClinTox	2	1478	ROC–AUC
MUV	17	93087	ROC–AUC
HIV	1	41127	ROC–AUC
BACE	1	1513	ROC–AUC
Esol	1	1128	RMSE
Freesolv	1	642	RMSE

As downstream tasks, we select 10 datasets from MoleculeNet^21^ including classification and regression shown as Table 1. Classification tasks include BBBP^26^, Tox21^27^, ToxCast^28^, SIDER^29^, ClinTox^30^, MUV^31^, HIV, BACE^32^ and regression tasks include Esol^33^, Freesolv^34^. Datasets which have multiple tasks are learned jointly in a single model.

- BBBP: Blood–brain barrier penetration. Prediction of the barrier permeability.
- Tox21: Qualitative toxicity measurements on 12 different targets, including nuclear receptors and stress response pathways.
- ToxCast: Data collection providing toxicology data based on in vitro high-throughput screening.
- SIDER: Drug side-effects into 27 system organ classes following MedDRA classification.
- ClinTox: It contains clinical trial toxicity and FDA approval status.
- MUV: Benchmark dataset from PubChem BioAssay. It contains 17 challenging tasks for validation of virtual screening techniques.
- HIV: The ability to inhibit HIV replication.
- BACE: Binding results for a set of inhibitors of human $\beta$-secretase 1.
- Esol: Water solubility data.
- Freesolv: Hydration free energy of small molecules in water.

In fine-tuning, we apply scaffold splitting into classification tasks and random splitting on regression tasks. Scaffold splitting is effective in performing out-of-distribution samples. Molecules are processed with the RDKit library. Our model has trained with Adam optimizer with a learning rate of {0.00001, 0.00005}. We use 15 epochs on classification and 40 epochs on regression. We use a batch size of {64, 128}. The split ratio of train/valid/test is 80%:10%:10%. We evaluate classification tasks using ROC–AUC and regression tasks using Root Mean Square Error (RMSE). ROC–AUC is a performance measurement metric for classification problems at various threshold settings. We take the average ROC–AUC on datasets that have multiple tasks. An early stopping process based on the best validation score is applied to all tasks. We record the average performance of 5 times.

## Results and discussion

We use the feature-engineered machine learning model and SMILES-based deep learning models as baselines. RandomForest^35^ is a traditional machine learning approach. We use Extended Connectivity Fingerprint (ECFP)^5^ and descriptors of molecules as input features. The set of descriptors contains lots of chemical features from molecules such as alogP, HBD, HBA, QED, Molecular Weights, Rotatable bonds, Number of Rings, Number of Aromatic Rings, Number of Atoms, Number of Heavy Atoms, Number of Bonds, Number of Valence Electrons, Partial Charge (min, max), Number of Aliphatic Carbocycles, Number of Aliphatic Heterocycles, Number of Aliphatic Rings, Number of Aromatic Carbocycles, Number of Aromatic Heterocycles, etc. As a pre-trained model, SMILES-BERT is used for comparisons. SMILES-BERT follows the concept of BERT and is pre-trained by masked token prediction. We also report the performance of non-pretrained models to evaluate the effect of pre-training methods.

**Table 2:** ROC–AUC (%) performance on classification tasks. The best score of each dataset is marked as bold.

	Pretrained	BBBP	Tox21	ToxCast	SIDER	ClinTox	MUV	HIV	BACE	Avg
RandomForest	No	$74.7 \pm 0.5$	$76.8\pm 0.1$	$65.3 \pm 0.2$	$64.8 \pm 0.6$	$78.5\pm 1.3$	$65.9\pm 2.0$	$77.8 \pm 1.1$	$83.0 \pm 0.6$	73.4
SMILES-BERT	No	$67.8\pm 0.8$	$72.4\pm 0.7$	$60.8\pm 0.3$	$58.2\pm 0.6$	$98.8\pm 0.0$	$60.6\pm 1.8$	$71.4\pm 1.6$	$76.4\pm 1.7$	70.8
CHEM-BERT	No	$66.5\pm 0.5$	$73.6\pm 0.6$	$61.4\pm 0.3$	$58.2\pm 0.5$	$98.6\pm 0.1$	$64.2\pm 0.7$	$72.7\pm 1.6$	$78.4\pm 0.6$	71.7
SMILES-BERT	Yes	$73.3\pm 1.0$	$75.8\pm 0.6$	$61.5\pm 0.9$	$61.8\pm 0.3$	$97.9\pm 0.8$	$71.1\pm 1.6$	$76.6\pm 1.1$	$80.0\pm 3.3$	74.8
CHEM-BERT	Yes	$72.4\pm 0.9$	$77.4 \pm 0.5$	$65.3 \pm 1.1$	$63.1\pm 0.6$	$99.0 \pm 0.3$	$73.2 \pm 1.7$	$77.6 \pm 1.6$	$82.0\pm 1.7$	**76.2**

**Table 3:** RMSE on regression tasks. The best score of each dataset is marked as bold.

	Pretrained	Esol	Freesolv	Avg
RandomForest	No	$1.279\pm 0.127$	$2.419\pm 0.293$	1.849
SMILES-BERT	No	$0.561\pm 0.029$	$1.256\pm 0.088$	0.908
CHEM-BERT	No	$0.544\pm 0.031$	$1.220\pm 0.075$	0.882
SMILES-BERT	Yes	$0.473\pm 0.052$	$0.810\pm 0.088$	0.641
CHEM-BERT	Yes	$0.451 \pm 0.033$	$0.755 \pm 0.078$	**0.603**

**Figure 3:**
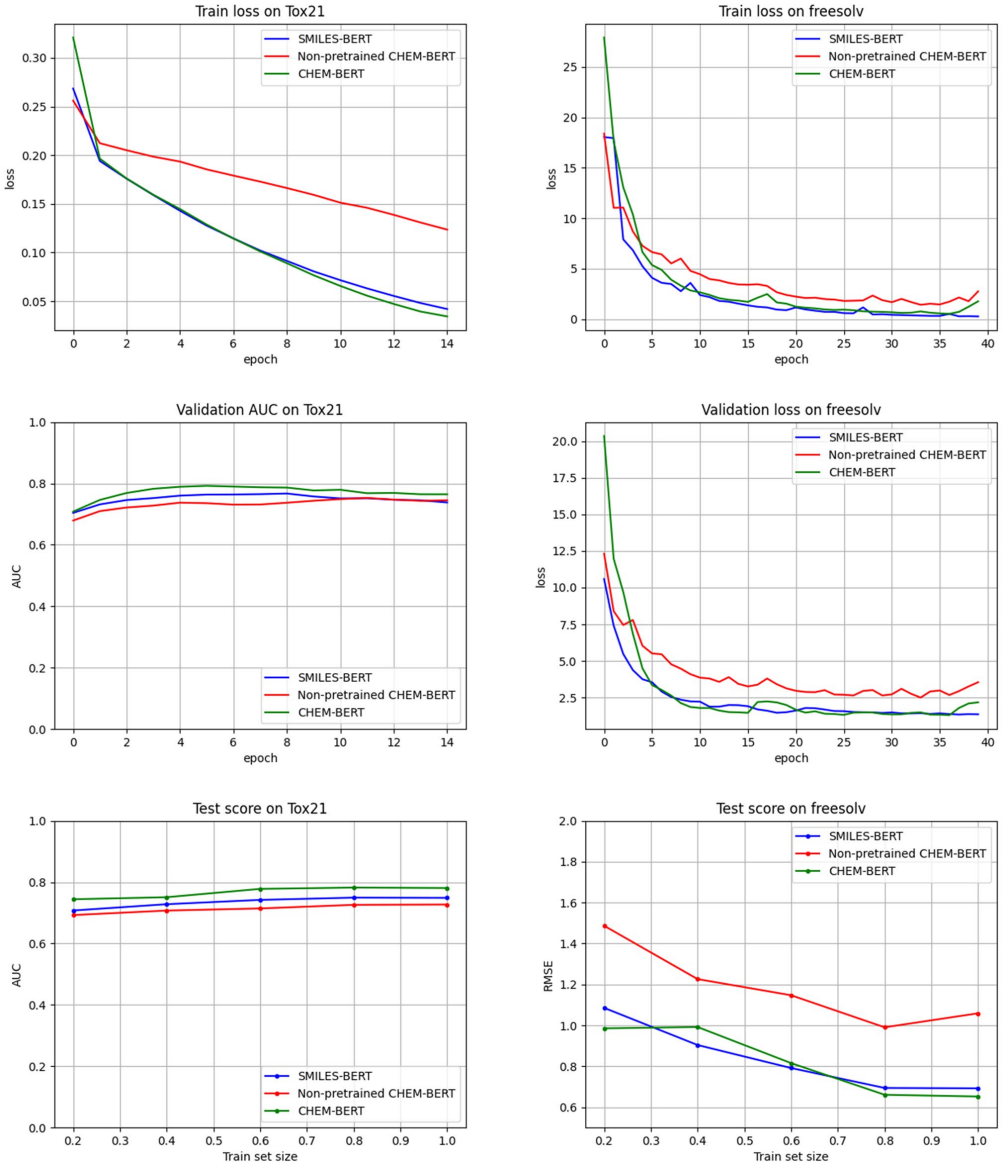
Train, validation, and test curves of Tox21 (classification) and Freesolv (regression). Pre-trained models converge faster than a non-pretrained model shown in train and validation curves. Test curves show model performance against different train sizes. CHEM-BERT performs better than others at almost all training points.

We report results on molecular properties predictions in Tables 2 and 3. In the case of RandomForest, it shows competitive results on classification tasks. We can infer that manually engineered features such as descriptors are still effective in training classification tasks. From that result, we judged that extracting the chemical properties of molecules is helpful in the training process. Therefore, we design the QED prediction task to train our model that can infer the chemical properties and utilize them in predicting. We also report results from the non-pretrained model of SMILES-BERT and CHEM-BERT. Significant performance improvements have been made to the overall downstream tasks by pre-training the model. Compared to SMILES-BERT, our model gives better performance on most downstream tasks with higher improvements. The result shows that our method learns chemical contexts of molecules effectively in pre-training. We also report the learning curves of pre-trained models in Fig. 3. Loss and ROC–AUC curves on classification and regression tasks are reported. Our model achieves a faster convergence speed on training and validation than other models. We also report the performance according to the size of the train set. Our model also achieves better performance on the test set than other models at almost all training points. As a result, our model shows the best average performance in both classification and regression.

**Table 4:** Comparison with a pre-training strategy used in GNN. ROC–AUC (%) on classification tasks and RMSE on regression tasks.

	BBBP	Tox21	ToxCast	SIDER	ClinTox	MUV	HIV	BACE	Esol	Freesolv
Pretrained-GIN	$68.7\pm 1.3$	$78.1 \pm 0.6$	$65.7 \pm 0.6$	$62.7\pm 0.8$	$72.6\pm 1.5$	$81.3 \pm 2.1$	$79.9 \pm 0.7$	$84.5 \pm 0.7$	$0.605\pm 0.064$	$1.112\pm 0.127$
CHEM-BERT	$72.4 \pm 0.9$	$77.4\pm 0.5$	$65.3 \pm 1.1$	$63.1 \pm 0.6$	$99.0 \pm 0.3$	$73.2\pm 1.7$	$77.6\pm 1.6$	$82.0\pm 1.7$	$0.451 \pm 0.033$	$0.755 \pm 0.078$

To evaluate whether the pre-training tasks are well designed, we compare our method with a pre-training strategy^20^ applied on GNN. It uses a base model as Graph Isomorphism Network (GIN)^36^. GIN is a kind of message-passing neural network that is implemented to conduct the Weisfeiler–Lehman (WL) graph isomorphism test. It can distinguish topological identical of graphs. Pre-trained GIN shows better performance than other pre-trained GNNs such as Graph Convolutional Networks. The main tasks of the pre-training method on GIN are supervised graph-level property prediction and graph structure prediction. Our method shows competitive performance compared with the GNN-based method shown in Table 4. Except for the MUV dataset, there is no model with a poor performance. Compared to other types of pre-training tasks, it is confirmed that our method is composed of well-designed tasks.

**Figure 4:**
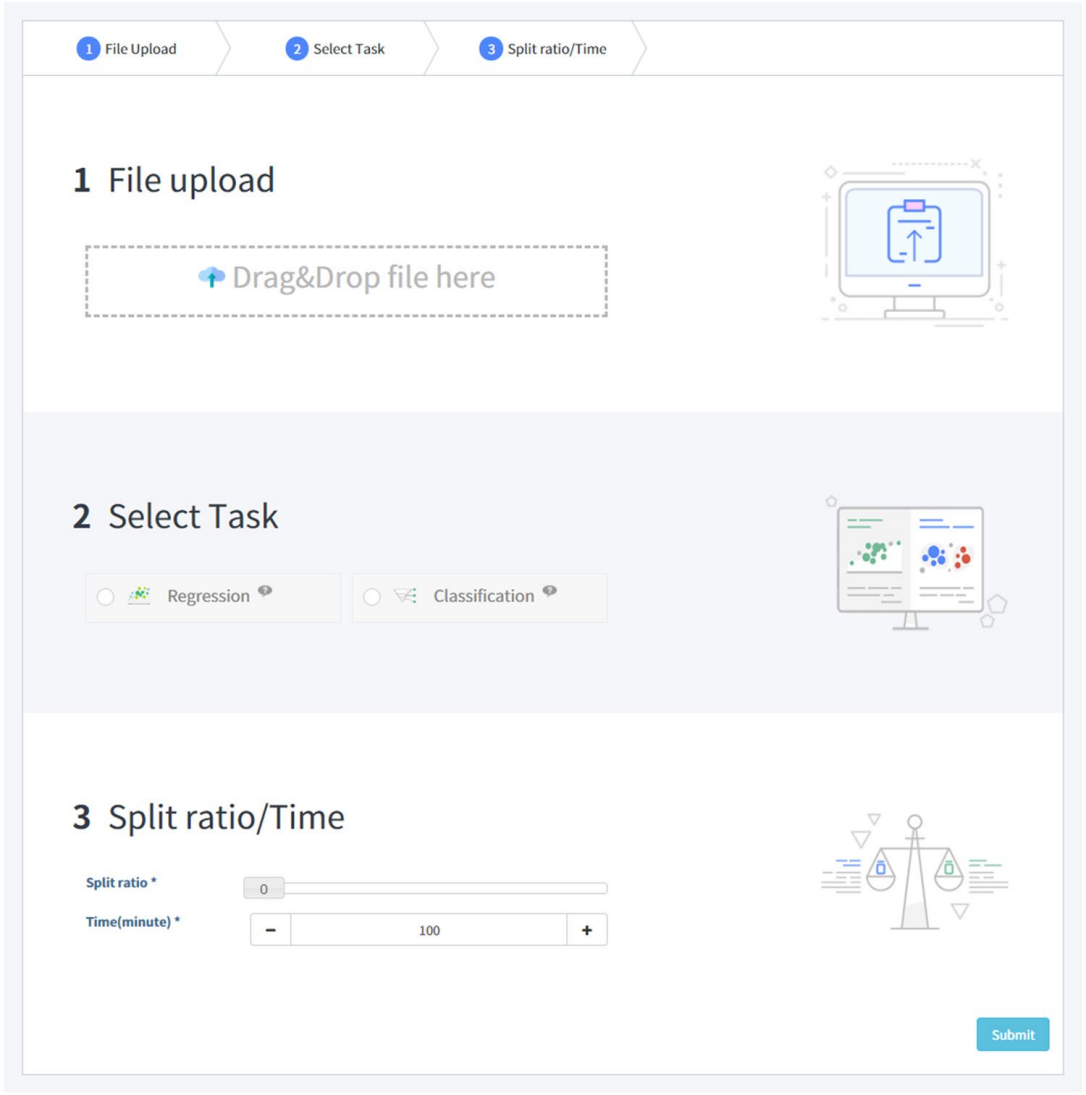
Job submission page of the fine-tuning service. Users upload their own dataset and select the type of task. There is no specific parameter selection for training.

**Figure 5:**
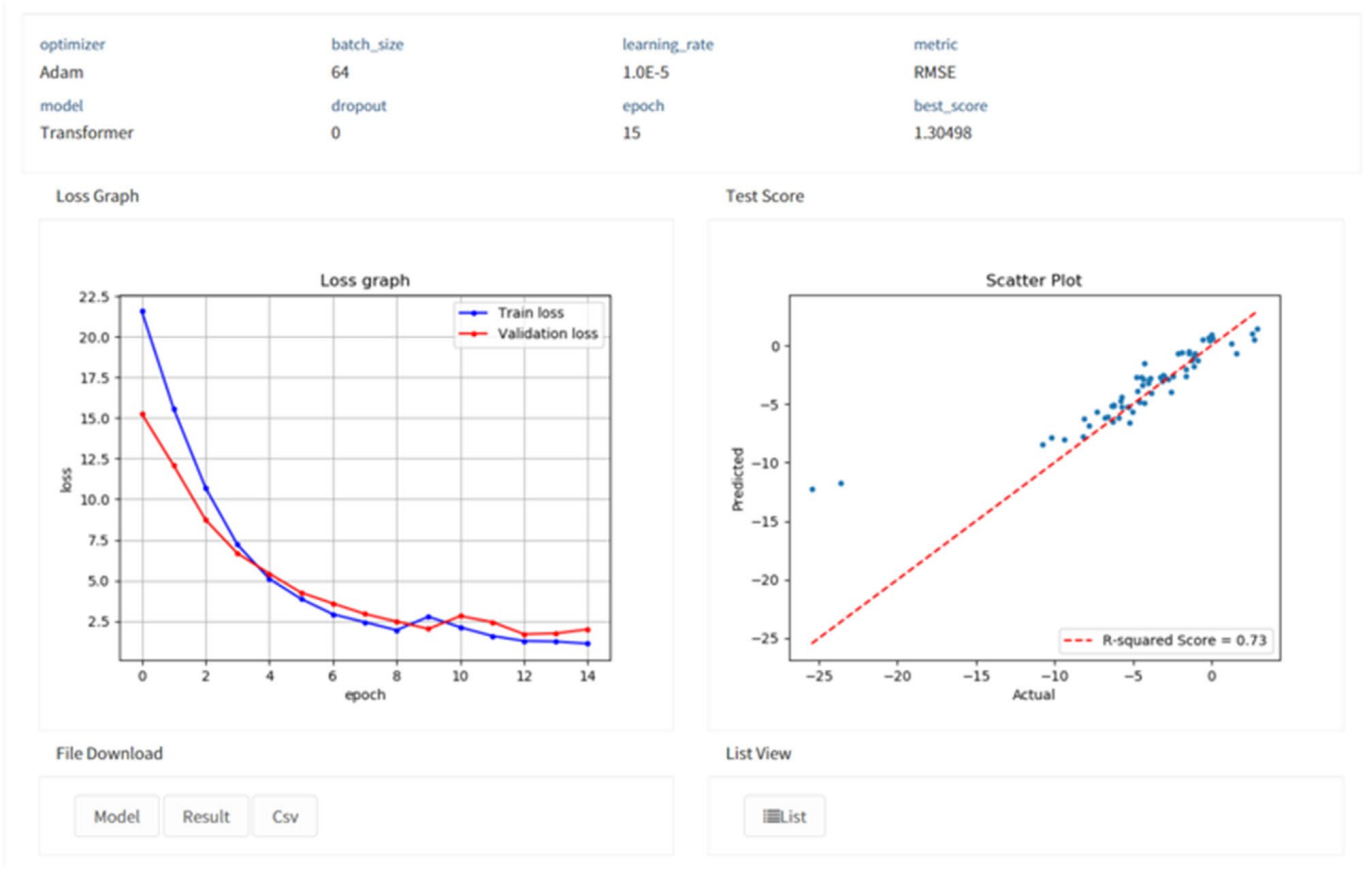
The result of the fine-tuning service. The result contains hyperparameters of the model, loss graph and performance on test set. Users can export the result and the model.

We develop the fine-tuning service based on our pre-trained model shown as Fig. 4. We design the service to utilize a characteristic of the pre-trained model that converges faster than a non-pretrained model. For using the service, users should prepare a dataset which contains SMILES and label of data in CSV format. Before job submission, users select the type of task, data split ratio, and time limitation. We minimize the optional parameters for fine-tuning to provide a user-friendly service. After job submission, users can get the result of training and performance on submitted data shown as Fig. 5. We expect three contributions from the fine-tuning service. First, users do not have to spend lots of time and cost to pre-train the model. By using the service, users can skip the pre-training stage. The service also contains the pre-processing logic. Therefore, the user does not have to design additional processing logic. Second, users do not have to design a model specifically and they can train various molecular datasets. Using the service, they can build the model without domain knowledge on data and machine learning. Finally, users can obtain stable and high performance. As seen from our experiment results, we constructed a generalized model that can perform various molecular properties predictions. They can easily access the service by submitting datasets and get reliable results. Users can start their new research or get insights about data by building their model via our service. We expect our service can lower the barrier of artificial intelligence for chemistry researchers.

## Conclusions

Our work suggests constructing a robust pre-trained model by effectively integrating various representations such as SMILES and an adjacency matrix from molecules. Based on the Transformer, we designed the matrix embedding layer for learning connectivity and added a QED prediction task in pre-training. Our method achieved better average performance than other models on downstream tasks and showed stable performance on various data. From the result, it has been shown that our model learns the general properties of molecules well. We also implemented the fine-tuning service to provide a user-friendly training environment with a pre-trained model. We expect users could save their effort and costs for constructing and training the prediction model. Users can easily train the model without hyperparameter-tuning and get the result from our service. We make one step forward on designing a novel representation of molecules and utilizing the model in various fields via the fine-tuning service. For future work, we plan to devise a representation that can import 3D structures and apply our service to drug discovery processes such as virtual screening and drug–target interaction.

## Data Availability

The datasets used in pre-training and evaluation are available at ZINC and MoleculeNet. The source code of our work is available at https://github.com/HyunSeobKim/CHEM-BERT.
